# Case report: ATIC-ALK fusion in infant-type hemispheric glioma and response to lorlatinib

**DOI:** 10.3389/fonc.2023.1123378

**Published:** 2023-02-24

**Authors:** Shubin W. Shahab, Matthew Schniederjan, Jose Velazquez Vega, Stephen Little, Andrew Reisner, Tobey MacDonald, Dolly Aguilera

**Affiliations:** ^1^Aflac Cancer and Blood Disorders Center, Children’s Healthcare of Atlanta, Atlanta, GA, United States; ^2^Department of Pediatrics, Emory University School of Medicine, Atlanta, GA, United States; ^3^Department of Pathology, Emory University School of Medicine, Atlanta, GA, United States; ^4^Children’s Healthcare of Atlanta, Atlanta, GA, United States; ^5^Department of Radiology, Emory University School of Medicine, Atlanta, GA, United States; ^6^Department of Neurosurgery, Emory University School of Medicine, Atlanta, GA, United States; ^7^Winship Cancer Institute, Atlanta, GA, United States

**Keywords:** infant-type hemispheric glioma, high grade glioma, ALK fusion, lorlatinib, case report

## Abstract

**Introduction:**

Infant type hemispheric gliomas are a rare tumor with unique molecular characteristics. In many cases these harbor mutations in receptor tyrosine kinase pathways and respond to targeted therapy. Here we describe the case of an infant with this type of tumor with a novel ATIC-ALK fusion that has responded dramatically to the ALK inhibitor lorlatinib, despite being refractory to standard chemotherapy.

**Case description:**

The infant was initially treated with standard chemotherapy and found to have an ATIC-ALK fusion. When surveillance imaging revealed progressive disease, the patient was switched to the ALK-inhibitor lorlatinib at 47 mg/m^2^/day. The patient demonstrated a significant clinical and radiographic response to the ALK inhibitor lorlatinib after just 3 months of treatment and a near complete response by 6 months of therapy.

**Conclusion:**

The ALK inhibitor lorlatinib is an effective targeted therapy in infant type hemispheric glioma patients harboring ATIC-ALK fusion.

## Introduction

Infantile high-grade gliomas (HGG) are rare entities with unique molecular biology. These tumors are now codified in the World Health Organization (WHO) classification of Central Nervous System Tumors as infant-type hemispheric gliomas (herein referred to as ITHG) without a specific corresponding grade (1). In contrast to their adult counterpart, ITHGs exhibit better outcomes. Prior studies have demonstrated that patients under 4 years with HGG can have distinct molecular rearrangements and fusions and tend to have a more favorable prognosis compared to older patients (2, 3). One of the genes commonly altered in ITHGs is anaplastic lymphoma kinase (*ALK*), which encodes a receptor tyrosine kinase (RTK) that is also often rearranged, amplified or mutated in pediatric neoplasms, including anaplastic large cell lymphoma (ALCL), inflammatory myofibroblastic tumor (IMT), rhabdomyosarcoma, glioma and neuroblastoma (2). In a case series of infants with HGGs, a large number of cases demonstrated gene fusions that can be targeted with novel therapies. Among these, MET fusions were present in 4 samples, NTRK1/2/3 in 21 samples, ROS fusions in 9 samples and ALK fusions in 31 samples, the most common among which was the PPP1CB-ALK fusion (3). While the 5-aminoimidazole-4-carboxamide ribonucleotide formyltransferase *(ATIC)-ALK* fusion has been described in literature, it is a rare fusion event and has never been described in an ITHG. Here, we describe the case of a patient with an ITHG diagnosed at 3 months of age and exhibiting an *ATIC-ALK* fusion, who despite progression on standard chemotherapy, has had an extremely good radiographic and clinical response to the ALK inhibitor lorlatinib.

## Case presentation

**Figure 1:**
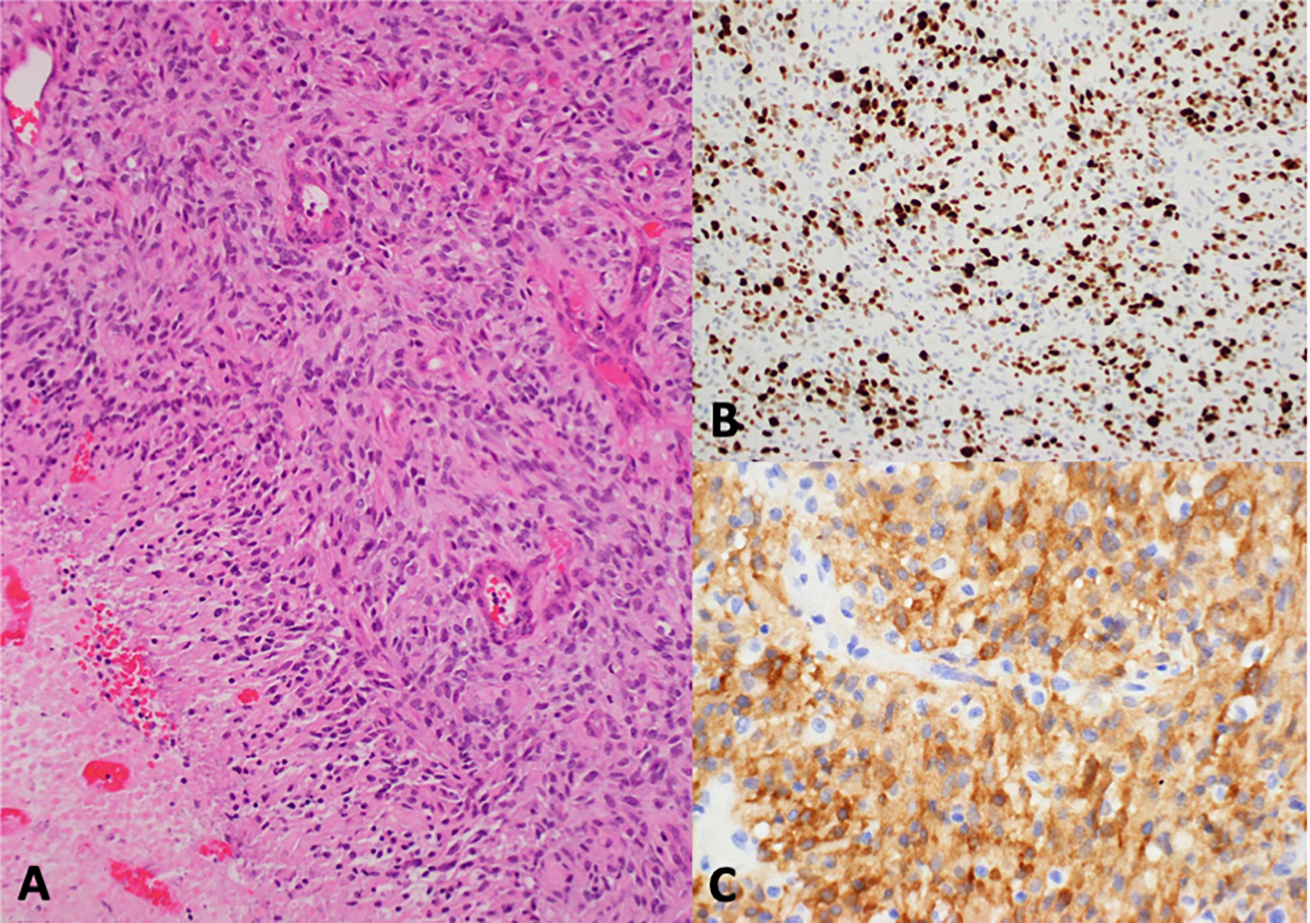
Histologic sections showed a highly cellular glial tumor with a solid growth pattern, brisk mitotic activity, microvascular proliferation, and palisading necrosis **(A)**. Immunohistochemistry revealed high Ki-67 expression **(B)** and strong cytoplasmic ALK expression **(C)**.

**Figure 2:**
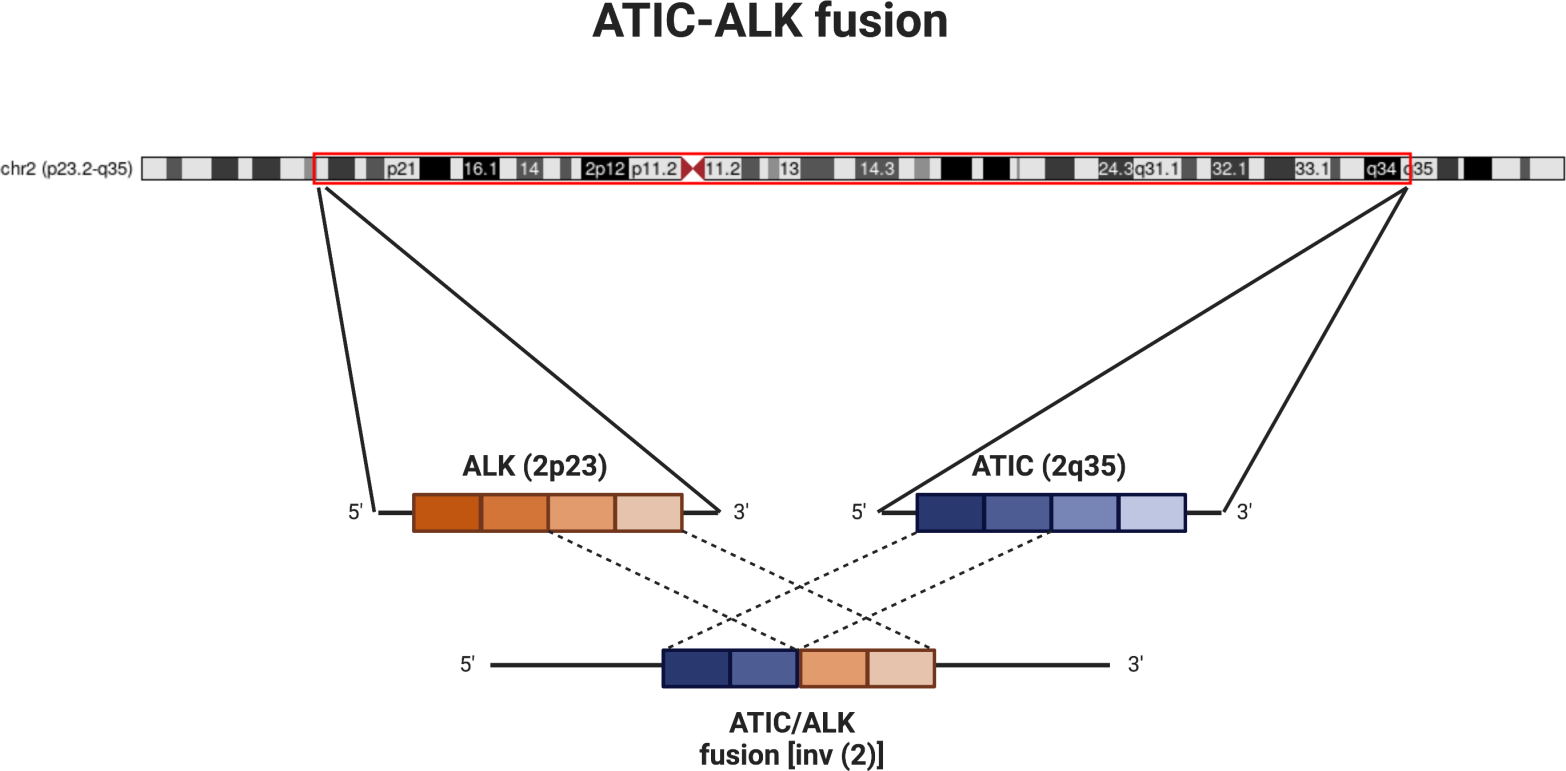
Schematic of ATIC-ALK fusion. Diagram showing chromosome 2 (courtesy of UCSC genome browser; http://genome.ucsc.edu (4); with approximate locations of ALK (2p23) and ATIC (2q35). The inversion and subsequent fusion between the 5’ end of ATIC to the 3’ end of ALK leads to activation of the ALK kinase. For our patient, the fusion is noted specifically between exon 7 of ATIC and exon 20 of ALK.

**Figure 3:**
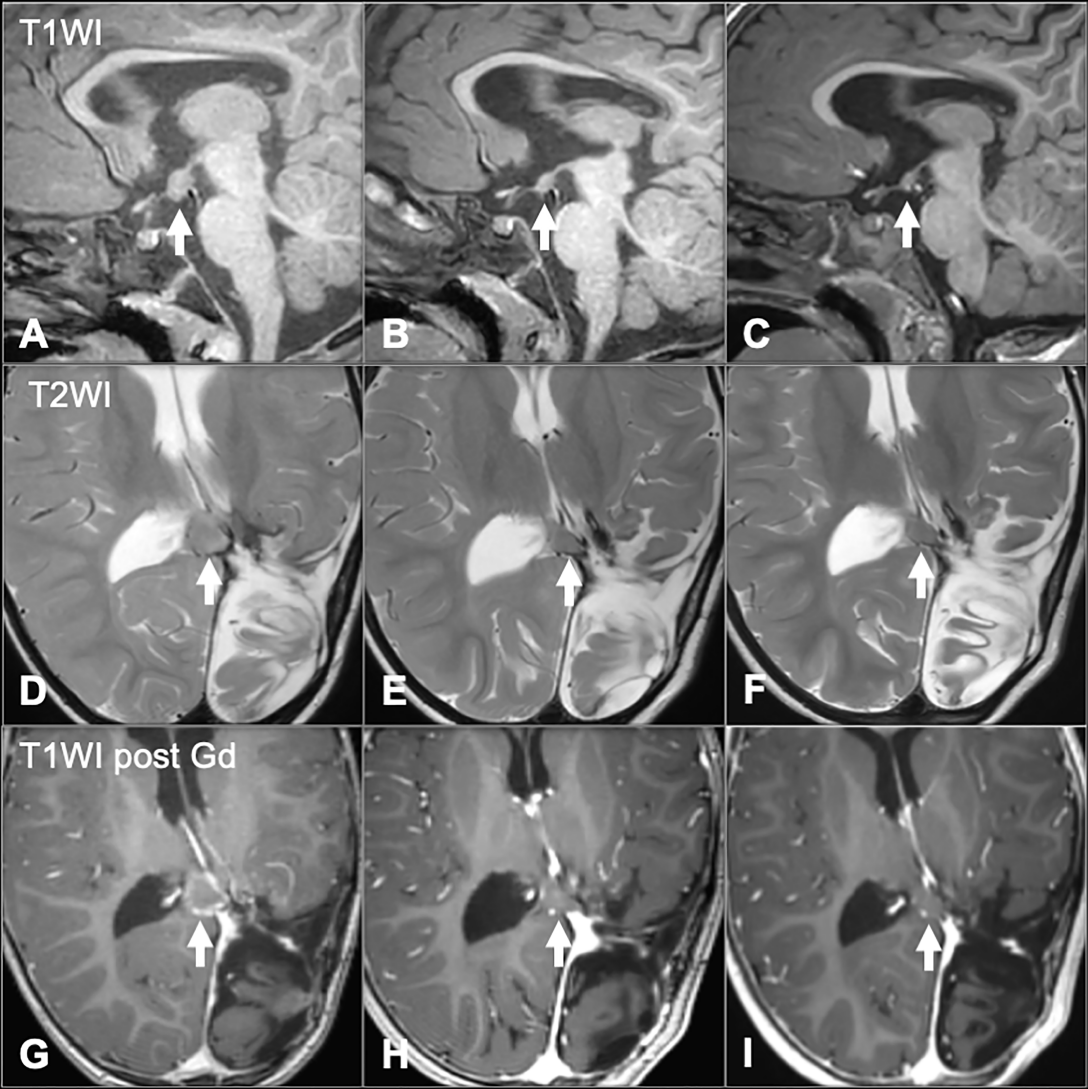
Pre **(A**, **D**, **G)**, Post 1 (10 weeks) **(B**, **E**, **H)** and Post 2 (6 months) **(C**, **F**, **I)** ALK inhibitor administration. Pre-treatment images demonstrate nodular lesions near the splenium of the corpus callosum (arrows in **D**, **G**) and along the floor of the third ventricle (arrow in **A**). Post-treatment images (Post 1) demonstrate substantial decrease in size of both lesions (arrows in **B**, **E**, **H**). Continued decrease in lesion size is seen on Post 2 images (arrows in **C**, **F**, **I**).

**Figure 4:**
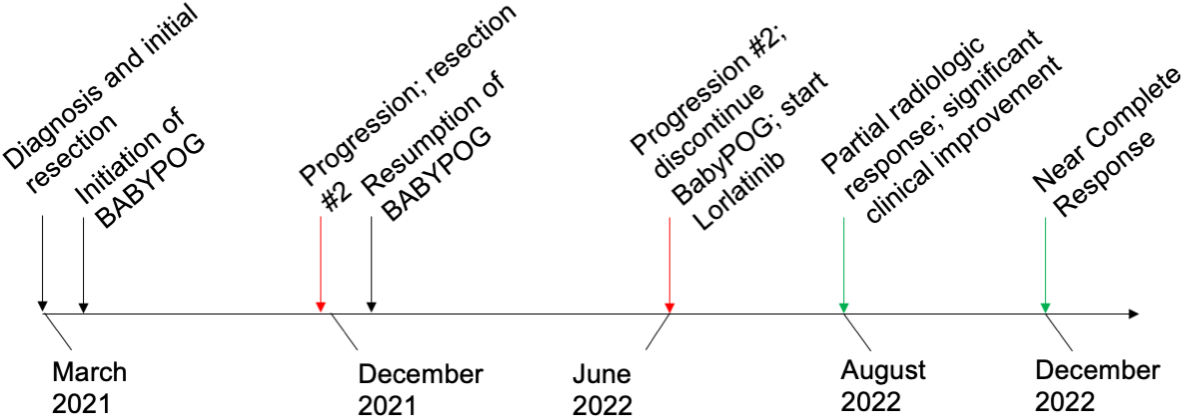
Timeline of major clinical events since diagnosis to present day.

A 3-month-old patient presented with poor head control, increased fatigue, right hemiparesis, feeding difficulties, decreased peripheral vision on the right side and progressively increasing head circumference. Emergency department evaluation showed bulging fontanelle, increased irritability and macrocephaly. A head CT revealed a large, left sided supratentorial mass with midline shift and hydrocephalus, requiring ventricular drain placement. MRI of the brain confirmed a large tumor centered in the posterior left cerebral hemisphere with associated severe mass effect, including subfalcine, uncal, and ascending transtentorial herniation. There was no evidence of metastatic disease in the brain or spine. The patient underwent a subtotal resection with significant blood loss intraoperatively requiring initiation of massive transfusion protocol. Pathology revealed a densely cellular HGG with focal pseudopalisading necrosis, gemistocytic morphology in a proportion of the tumor as well as elevated mitotic activity. A panel of immunohistochemistry supported the diagnosis of HGG (**Figure 1**). Furthermore, DNA methylation analysis classified the tumor as an ITHG. The sarcoma fusion panel (Archer FusionPlex) revealed fusion between *ATIC* (exon 7) and *ALK* (exon 20) (**Figure 2**). Additional (Ashion) testing through the precision medicine program at our center confirmed the *ATIC-ALK* fusion at both the DNA and RNA level. There was no evidence of germline mutations. Initial treatment with multiagent chemotherapy per the Baby POG protocol (5) induced a partial tumor response; however, after 7 cycles of this chemotherapy regimen, routine surveillance brain MRI demonstrated progressive disease. The patient underwent a second subtotal resection and resumed Baby POG chemotherapy for an additional 8 months. Due to further tumor progression in multiple areas of the resection cavity on brain MRI, and considering the result of tumor sequencing, the patient’s treatment was then switched to the ALK inhibitor lorlatinib at a dose of 47 mg/m^2^/day *via* gastrostomy tube. Subsequent brain MR imaging after 10 weeks of daily lorlatinib treatment revealed a partial response in the largest residual tumor lesion and complete response on the smaller tumor nodules (**Figure 3**) and 6 months after starting lorlatinib, near complete response on all the residual tumor nodules (**Figure 3**). At the time of this report, the patient continues on daily lorlatinib therapy (See timeline **Figure 4**). The only toxicities to date per common terminology criteria for adverse events (CTCAE, version 5.0) have been Grade 1 hypercholesterolemia, Grade 1 triglyceridemia and Grade 2 weight gain. No neurological toxicities have been observed. The clinical response has been remarkable with resolution of hemiparesis, improvement of gait, speech and language, and stabilization of visual deficits.

## Caregiver perspective

The mother of the child shared the following with the primary oncologist: “Our quality of life has improved significantly since switching to lorlatinib. Our child has had no admissions for fever or neutropenia, no need for transfusion of blood products, and has experienced no nausea or vomiting or additional hearing loss. Her appetite is back to normal, she no longer needs g-tube feeds, and is now eating all meals by mouth. She has also achieved more developmental milestones since starting lorlatinib.

Also, we have had fewer clinic visits and lab draws as she has needed less monitoring, going from weekly visits during standard chemotherapy to once every 6 weeks after starting lorlatinib. The central line has been removed. Our child is back at day care. “

## Discussion

ALK is a tyrosine kinase that can often be aberrantly expressed in cancers due to rearrangements or mutations and amplification events. In ALCL, where ALK oncogenic fusions were first described, the most frequent fusion partner is nucleolar protein nucleophosmin (NPM) and accounts for 40-60% of cases. Overall, ALK fusions occur in up to 90% of ALCLs in children, and up to 50% of adult cases (6). Other common fusion partners for ALK include TPM3, TPM4, CLTC and EML4, which are found across different tumor histologies including ALCL, IMT, papillary thyroid cancer, and renal cell carcinoma (2). The *ATIC-ALK* fusion is a rarer occurrence; The *ATIC* gene encodes a bifunctional enzyme involved in purine biosynthesis. During the rearrangement the portion of *ATIC* gene encoding the amino terminus is fused with the portion of *ALK* gene encoding its carboxy terminus, the product of which leads to a constitutively active ALK tyrosine kinase (7) (**Figure 2**).

The *ATIC-ALK* fusion has been reported in a variety of cancers including in IMT, ALCL, and NSCLC (2, 6–14); overall, it still accounts for only 0.1% of reported AACR Genie cases of ALK fusions (15). Thus, to our knowledge, this is the first report of a patient with an ITHG with the *ATIC-ALK* fusion treated with targeted therapy using an ALK inhibitor, even though other groups have reported the use of lorlatinib for ITHG with other ALK fusions (16).

ITHGs are unique in that most of these tumors harbor alterations in RTK oncogenes (up to 80% in one study (17)). It is possible that the presence of these RTK alterations may drive the biology behind the favorable prognosis seen in infant HGG (17) and may display maturation/differentiation following treatment.

ALK fusions in infant brain tumors have been reported (3, 18–21). In a large multi-institutional international cohort study (17) of infant gliomas, the reported ALK fusions were CCDC88A-ALK, EML4-ALK, PPP1CB-ALK and KTN1-ALK. In this study infants with ALK fusion positive HGGs had a worse outcome compared to ALK fusion positive low grade glioma (LGG), with only 57.1% alive at 3 years of median follow up. In another large study CLIP2-ALK, HIP1-ALK, MAD1L1-ALK, MAP2-ALK, MSI2-ALK, PRKAR2A-ALK, SPECC1L-ALK, SYNDIG1L-ALK and ZC3H7A-ALK fusions were reported (3). In one study, an infant with HGG and PPP1CB-ALK fusion had a complete resection and has not had progression of the tumor 3 years since diagnosis without additional therapy (22). On the other hand, more aggressive courses have been reported with ALK fusion positive infant HGG, and in some cases, response to targeted therapy with ALK inhibitors has been documented (16). ALK inhibitors have also demonstrated efficacy in patients with metastatic NSCLC (non-small cell lung cancer) to the brain, as well as in neuroblastoma and there are several clinical trials that are currently looking into the efficacy of ALK inhibitors in both CNS and non-CNS tumors (NCT03236675, NCT04774718, NCT02201992, NCT02568267, NCT03052608, NCT04589845, NCT02693535, NCT04541407, NCT04094610, NCT02650401).

Lorlatinib is a 3^rd^ generation ALK inhibitor with improved CNS penetration compared to earlier generations, and may have some neurological toxicities including peripheral neuropathy, mental status and mood changes (23). In a clinical trial for adult patients with advanced ALK-positive lung cancer the 12-month event-free survival was 78% in the lorlatinib arm vs. 39% in the crizotinib arm. Additionally, the responses were significantly better with lorlatinib for subjects with intracranial metastatic disease, a subset in which 71% in the lorlatinib group achieved complete response in the brain (as opposed to 8% for crizotinib) (24).

At this time, the optimal pediatric dose, duration of treatment, or anticipated long term toxicities of using lorlatinib in children, let alone in infants, are not known. Our patient has so far experienced the most common toxicities of hypercholesterolemia, hypertriglyceridemia and weight gain, although some neurocognitive effects may be difficult to assess at this age. In a previously published case report of lorlatinib use in a child with HGG, the patient was treated with 95 mg/m^2^/day (twice the dose used in our patient) with similar toxicity profile (16). That patient received treatment for 8 months (with a 20% dose reduction due to weight gain) and then therapy was discontinued for a period before having to be resumed due to relapse. Another case of a young child with a recurrent infant-type hemispheric glioma with the *ZNF397-ALK* fusion has recently been published (25). In that study the patient received lorlatinib at 95 mg/m2/day as well. Significant complications were reported including weight gain greater than 130% from baseline. A dose reduction of 50% of lorlatinib was started, and while weight gain slowed, it did not stop. Eventually lorlatinib was discontinued after more than 1 year of treatment. Prior use of lorlatinib in children also included a phase I trial to evaluate doses on relapsed or recurrent neuroblastoma patients with ALK mutations/amplification who might have had prior exposure to other ALK inhibitors (26). In that study 5 dose levels (45, 60, 75, 95, 115 mg/m2/day) were evaluated in pediatric patients. Dose limiting toxicity (DLT) was the primary endpoint on the first 28 days and neurocognitive toxicity within 54 days of starting drug. Of note the youngest patient getting lorlatinib on this trial reported was 2 years old. No DLT was observed at the lowest 3 dose levels. At a dose of 95mg/m2/day 50% enrolled patients (5/10) had DLT’s. At 115/mg/m2/day 33% of enrolled patients (1/3; with expansion ongoing at the time of abstract publication) had a DLT of grade 3 diarrhea. Overall weight gain, hyperlipidemia, concentration/memory impairment, peripheral neuropathy, and peripheral edema were the most common adverse events reported. Interestingly, responses were seen early (median of 2 courses) and across dose levels.

Given that responses were seen at the lowest dose level, we selected this dose to start the treatment of our patient. Our patient has only experienced Grade 1 hypercholesterolemia, Grade 1 triglyceridemia and Grade 2 weight gain to date and as highlighted in the caregiver perspective has had significant improvement in quality of life. While targeted therapy clearly has a role in the relapsed/refractory setting, it remains to be seen whether this approach will be widely adopted as 1^st^ line. In patients with ALK-rearranged non-small cell lung cancer targeted therapy appears to be superior than chemotherapy only regimens (27). Recently a meta-analysis investigated whether ALK-inhibitor therapy should be used first line in ALK-positive lung cancer patients who were also given chemotherapy (28). The authors noted that while there was an improvement in progression free survival, there was no significant improvement in overall survival. We recognize that ALK-positive lung cancer is a very different disease in a significantly older age group compared to ALK-positive infantile high-grade glioma and therefore, the outcomes may be very different. Furthermore, as with any case report, our observations may have been influenced by our subjective biases and may not be generalizable to other cases of high-grade glioma or even infants with IHTG. Thus, more studies are clearly needed. However, given the rarity of these fusions and the age of many of these patients, accruing enough patients for clinical trials may be difficult, although some data may be extrapolated from non-CNS studies or pathology agnostic trials in older patients.

This case highlights the importance of molecular information directing therapy in the clinical setting. We initially chose to treat our patient with standard chemotherapy per Baby POG after discussion within our group and with the parents. Specifically, the age of the child and the lack of data published in children less than 2 years of age were driving factors behind our decision. Additionally, the potential for development of resistance with prolonged exposure to this agent is also a consideration. Molecular profiling at the time of progression may help in our understanding of the pathways to resistance development and help find an alternative treatment. While there are no established combination treatment regimens with ALK inhibitors, several clinical trials are investigating rational strategies of combining ALK inhibitors with other agents.

## Data availability statement

The original contributions presented in the study are included in the article/supplementary material. Further inquiries can be directed to the corresponding author.

## Ethics statement

Ethical review and approval were not required for the study on human participants in accordance with the local legislation and institutional requirements. Written informed consent to participate in this study was provided by the participants’ legal guardian/next of kin. Written informed consent was obtained from the minor(s)’ legal guardian/next of kin for the publication of any potentially identifiable images or data included in this article.

## Author contributions

SS, TM and DA contributed to the study conception and design. Sample collection was performed by AR. Imaging analysis and interpretation were performed by SL. Histopathological slide preparation and interpretation were performed by MS and JV. The first draft of the manuscript was written by SS and all authors commented on previous versions of the manuscript. All authors contributed to the article and approved the submitted version.
